# Root growth in field-grown winter wheat: Some effects of soil conditions, season and genotype

**DOI:** 10.1016/j.eja.2017.09.014

**Published:** 2017-11

**Authors:** L. Hodgkinson, I.C. Dodd, A. Binley, R.W. Ashton, R.P. White, C.W. Watts, W.R. Whalley

**Affiliations:** a Lancaster Environment Centre, Lancaster University, LA1 4YQ, United Kingdom; b Rothamsted Research, Harpenden, Hertfordshire, AL5 2JQ, United Kingdom

**Keywords:** Wheat roots, Soil structure, Penetrometer resistance, Genotypic effects

## Abstract

This work compared root length distributions of different winter wheat genotypes with soil physical measurements, in attempting to explain the relationship between root length density and soil depth. Field experiments were set up to compare the growth of various wheat lines, including near isogenic lines (Rht-B1a Tall NIL and Rht-B1c Dwarf NIL) and wheat lines grown commercially (cv. Battalion, Hystar Hybrid, Istabraq, and Robigus). Experiments occurred in two successive years under rain fed conditions. Soil water content, temperature and penetrometer resistance profiles were measured, and soil cores taken to estimate vertical profiles of pore distribution, and root number with the core-break method and by root washing. Root length distributions differed substantially between years. Wetter soil in 2014/2015 was associated with shallower roots. Although there was no genotypic effect in 2014/2015, in 2013/2014 the dwarf wheat had the most roots at depth. In the shallower layers, some wheat lines, especially Battalion, seemed better at penetrating non-structured soil. The increase in penetrometer resistance with depth was a putative explanation for the rapid decrease in root length density with depth. Differences between the two years in root profiles were greater than those due to genotype, suggesting that comparisons of different genotypic effects need to take account of different soil conditions and seasonal differences. We also demonstrate that high yields are not necessarily linked to resource acquisition, which did not seem to be limiting in the low yielding dwarf NIL.

## Highlights

• We present evidence to support the hypothesis that the general and well-documented shape of the relationship between root length density and soil depth in UK grown winter wheat is related to the increase in soil strength with depth.
• Effects of the soil environment on root length distribution were greater than genetic effects and this was most likely related to soil saturation.
• In a dry season, there was genotypic variation in rooting depth.
• Greater root length at depth in the dwarf NILs suggests that deep rooting is not simply related to plant height.

## 1 Introduction

Wheat (*Triticum aestivum* L.) is a nutritionally and economically important crop grown in countries all around the world. The 2014/15 growing season produced 729.5 million tonnes globally, making it the second most produced crop worldwide, after maize (*Zea mays* L.) (FAO, 2016). The United Kingdom (UK), while having a relatively small agricultural land area compared to the main wheat producers, has some of the highest wheat yields of all countries, reaching a new world record in 2015 of 16.5 t/ha (Guinness World Records, 2015). In contrast, the global average wheat yield is a little under 3.1 t/ha (FAO, 2016). The relatively high yields in the UK can mainly be attributed to a mild climate where rainfall is distributed evenly through the year. However, in the UK, yields of winter wheat can be restricted by water availability (Dodd et al., 2011). Even in dry summers, water is available at depths as shallow as 60 cm at relatively high matric potentials (Whalley et al., 2007, Whalley et al., 2008), which has not been accessed by roots. Since water use (transpiration) is linearly related to crop yield (Passioura, 1977), this represents an untapped resource that might be usefully exploited to increase wheat yields.

The inability of roots to access water is commonly attributed to a low root length density at depth (Gregory et al., 1978a, Gregory et al., 1978b). For this reason, rooting depth of wheat in the UK has been of considerable interest (e.g. Lupton et al., 1974, Gregory et al., 1978a, Barraclough and Leigh, 1984, White et al., 2015). Lupton et al. (1974) found little difference between the depth of roots of tall wheats in comparison with semi-dwarf wheats which had recently been introduced to the UK. However, within wheats that are currently grown commercially in the UK, there is recent evidence that some lines are more effective at accessing deep water than others, although differences in water uptake at depth were not sufficiently large or consistent to identify extreme performers with any certainty (Ober et al., 2014). This may be partly due the impact of management on rooting depth. For example, sowing date can have a large impact on both the amount and depth of the roots, since total root mass was closely correlated with the accumulation of thermal time (Barraclough and Leigh, 1984). Early sowing led to deeper roots, especially until early spring (March), although the rooting depth was similar between early and late sown wheat thereafter. Taken together, these results indicate limited genetic differences in wheat root distribution with depth in the soil profile under UK conditions. Similarly, a comparison of different wheat lines at two different field sites in Australia found little effect of genotype in determining rooting depth, the amount of shallow roots or the amount of deeper roots (Wasson et al., 2014). The field sites (i.e. soil type) had the greatest effect on the distribution of roots with depth, with one of the sites encouraging a much greater root length density at depths shallower than approximately 1 m in all of the wheat lines.

In the field, deep roots are almost exclusively found in pre-existing pores (White and Kirkegaard, 2010), thus deep rooting is likely to be largely determined by the quantity of deep pores. While White and Kirkegaard studied root growth in a very different environment and soil in comparison with those found in the UK, Gao et al. (2016) have argued that their observation that deep roots are mainly found in pores is the general-case. Gao et al. (2016) suggested that increases in soil strength with depth may be responsible for confining roots to pores, especially when penetrometer resistances in the bulk soil are much greater than 2.5 MPa. In field studies, root length density decreases exponentially with depth (e.g. Gerwitz and Page, 1974, Fan et al., 2016), which contrasts to many laboratory experiments with re-packed soils (e.g. Manschadi et al., 2008, Jin et al., 2015, Gao et al., 2016), where there is relatively high root density at depth and a less noticeable exponential decrease of root length density with depth. Thus pore distribution with depth may explain the limited genetic differences in wheat root distribution with depth, but this has received little attention under UK conditions, especially with respect to deep roots.

This paper has two main goals. First, we compared root length density with the quantity of pores > 0.7 mm in diameter. While root length distributions of field grown wheat have been reported (e.g. Gregory et al., 1978a, Barraclough and Leigh, 1984, White et al., 2015) and they conform to the empirical root length density distribution of Gerwitz and Page (1974), they have not been compared with soil structural and physical characteristics. Indeed, Rich and Watt (2013) note that few field studies report both root and soil conditions. Second, we verify if changes in root length density with depth are genetically determined, by comparing tall and dwarf NILs (Rht-B1a Mercia (*Tall*), and Rht-B1c Mercia (*Dwarf*)) as well as wheat cultivars commercially grown in the UK. We report on measurements made in two successive seasons on adjacent fields with a similar soil. We discuss the effects of soil structure, genotype and season on the distribution of roots with depth.

## 2 Materials and methods

### 2.1 Experimental sites

Experiments were conducted on neighbouring Broadmead (2014) and Warren Field (2015) sites at Woburn Experimental Farm, Bedfordshire, UK (52°01′11.2N”0°35′30.4″W). At both sites, soil in the 0–40 cm layer was a silt-clay loam. The vertical gradient in texture, to a depth of 1 m, is negligible on Broadmead. However, on Warren Field there was sand at depths deeper than 40 cm. The differences in soil texture with depth were observed from 1 m long cores taken to measure rooting density (see below). On both sites the surface layer (approximately 30 cm) has more organic matter content and is less dense. Soil properties are summarized in Table 1. The soil profile on Broadmead is consistent with description of a soil profile by Weir et al. (1984) that should be expected to produce high yields of winter wheat.

**Table 1:** Description of the topsoil (0–40 cm below the surface) properties of Woburn Experimental Field Station, Bedfordshire, UK.

Property	Units	
Location	Latitude	5201′06′’N
	Longitude	0035′30′’W
Soil type	SSEW group	Typical alluvial Gley soil
	SSEW series	Eversley
	FAO	Fluvisol
Sand (2000–65 μm) Surface soil	g g^−1^ dry soil	0.538
Silt (63–2 μm)	g g^−1^ dry soil	0.203
Clay (<2 μm)	g g^−1^ dry soil	0.260
Texture	SSEW class	Sandy clay loam
Particle density	g cm^−3^	2.587
Organic matter	g g^−1^ dry soil	0.038

### 2.2 Field management

For both experiments, the sites were prepared by cultivation with a mouldboard plough, to a depth of 23 cm, and intensive cultivation approaches (i.e. power harrow) to produce a seedbed. Both fields were drained by tile drains. The field sites were sown in the same manner in both years, with a plot drill: 96 separate 9 m x 1.8 m plots, divided into four fully randomised blocks, with each block containing 23 plots of different wheat cultivars and one fallow plot. The experiment is also described by Whalley et al. (2017). Cultivars and fallow plots were randomly arranged within each block.

The plots were sown on 10/10/2013 in 2013/14 and 26/09/2014 in 2014/15. The field sites were rain fed with no additional irrigation. Soil moisture measurements were taken and soil cores were collected approximately 1 m from the end of each specific plot.

### 2.3 Wheat genotypes

Of the 23 available genotypes, five were selected for soil coring in 2014, and six in 2015, based on previous laboratory phenotyping experiments (Whalley et al., 2013). The 2014 genotypes were Battalion (*Bat*), Hystar Hybrid (*Hys*), Rht-B1c Dwarf Mercia (*Dwarf*), Rht-B1a Mercia (*Tall*), and Robigus (*Rob*). Rht-B1c Dwarf Mercia (*Dwarf*) and Rht-B1a Mercia (*Tall*) were near isogenic. The 2015 genotypes were the same as for the previous year, with the addition of Istabraq (*Ist*). We selected genotypes on the basis of contrasting rooting behaviour in laboratory experiments (unpublished data).

### 2.4 Field measurements

Neutron probe (CPI HydroProbe model 503TDR) readings were taken in the field at approximately monthly intervals. Aluminium access tubes were installed approximately 1 m from the end of selected plots and measurements were made at depths of 0.10, 0.25, 0.50, 0.75, 1.00, 1.25 and 1.45 m. Soil strength was measured by taking readings using a soil penetrometer, in both years (Whalley et al., 2008, Whalley et al., 2017). Where possible penetrometer strength profiles were taken to a depth of 52.5 cm. We used a penetrometer with a cone 9.45 mm in diameter at the base with a 30° semi-angle. Atmospheric conditions and rainfall were measured and recorded by a weather station on the experimental farm. Leaf area index was periodically measured witha ceptometer (Delta-T Devices, Burwell, Cambridge, UK) during the growingseason. Crop height was measured with meter ruler. At harvest, grain yield was measuredwith a plot combine harvester.

### 2.5 Soil cores to estimate rooting

Cylindrical soil cores were taken from the Broadmead plots between 03/06/2014 and 13/06/2014 and from the Warren Field plots between 25/06/2015 and 03/07/2015 using a soil column cylinder auger (Van Walt Ltd, Surrey, UK). The cores were 100 cm long and 9 cm in diameter. They were extracted approximately 1 m in from the end of the wheat plots at the end opposite to the one with the neutron probe access tube. In 2014 we took one core from three of the blocks for each genotype of interest. In 2015 four cores were taken for each genotype, one from each block. Once extracted, the cores were stored inside two 105 cm lengths of polyethylene guttering, wrapped in a black polyethylene bag, and stored at 4 °C at Rothamsted Research, Harpenden, UK, until analysis.

Cores were divided into five sections, each approximately 20 cm in length. These sub-cores were then broken approximately 5 cm from both ends to reveal fresh faces exactly as described by White and Kirkegaard (2010), such that the revealed faces would have been 10 cm apart in the original core.

The core faces were viewed at 3.95 x magnification and imaged using a Leica M205 FA stereomicroscope (Leica Microsystems), and Leica Application Suite Advanced Fluorescence (LAF AF) software (version 2.6.0, Leica Microsystems). Each entire face was photographed six times, to ensure complete coverage at 3.95 x magnification. The images were 1.4 MP in size with a resolution of 37.8 pixels per cm. When a whole face was not recovered (the cores were sometimes stony and crumbly, particularly at depths below 60 cm), then fewer images were recorded, but the entirety of the available face was photographed. All photographs were exported as TIF files to Adobe Photoshop CS5.1.

To determine root penetration through each face, 10 sections of 1 cm^2^ size were selected by overlaying 2 mm gridlines on the images from that face using Adobe Photoshop CS5.1., and using randomly generated coordinates to identify the 10 squares for analysis. The coordinates were generated using the RANDBETWEEN function in MS Excel 2010. The images were manually compared where the coordinates generated may have caused possible overlap, and when overlaps were identified, the second image was discarded and another 1 cm^2^ section chosen through a newly generated pair of coordinates. The numbers of roots and empty pores visible per individual 1 cm^2^ were recorded in an Excel 2010 spreadsheet before being exported for statistical analysis. For this study, pores were defined as a visible airspace in and below the broken face of the soil core, with a diameter ≥ 0.7 mm. Where the airspace was not circular in form (e.g. a crack within the core), then it was considered a pore if the narrowest point of the airspace was ≥ 0.7 mm across. Using this reasoning, cracks in the core and other large airspaces were also considered pores.

After the core break and photography procedures were complete, each 10 cm subsection of core was stored in a polythene bag and frozen at −23 °C. The subsections were then defrosted and the soil and debris washed out through a 0.5 mm sieve to retrieve as many root fragments as possible. These root fragments were then scanned on a flatbed scanner and analysed using WINRhizo (Pro Arabidopsis, version 2008a, Regent Instruments Inc., Quebec, Canada).

### 2.6 Statistical analysis

All experimental data were analysed with GenStat v16 (www.vsni.co.uk). In each of the experimental years (2013/14 and 2014/15), 23 lines of wheat and a fallow plot were set out in a fully randomised complete block in four blocks, although we only made root measurements on a subset of these lines, as explained above. A different randomisation scheme was used in each year. The block structure, block/plots, was used for the statistical analyses with a treatment structure of “wheat line” for yield measurements and block/plots/depth was used with the treatment structure “wheat line*depth” for the penetrometer and root measurements. Penetrometer data was analysed with REML (residual maximum likelihood), but these data required square root transformation to stabilize the variance with spline models to account for the profile with depth. For ease of comparison with other published data, penetrometer data were plotted on the natural scale, while plotting the standard error of differences (SED) obtained from the transformed data was not possible. Similarly, the numbers of roots were transformed using square roots and the profiles modelled with regression (depth being treated as a variable) for a linear trend and spline models to represent the non-linear departure from the linear response. When fitting the spline function, a linear trend was used to explain the decrease in root numbers with depth and the spline curve was superimposed on the linear trend to account for the nonlinear nature of the root count with depth. Thus, the slopes of the linear trend were compared to determine if there were any significant differences because this represents an interaction between wheat line and depth. Given the low numbers of roots at depth, a variance determined from the surface layers, was imposed on the deeper layers. The spline fits were compared using the Wald statistic from REML, and depth was treated as variable (see Appendix A). Yield data was analysed with ANOVA.

## 3 Results

### 3.1 Soil penetrometer resistance

**Fig. 1:**
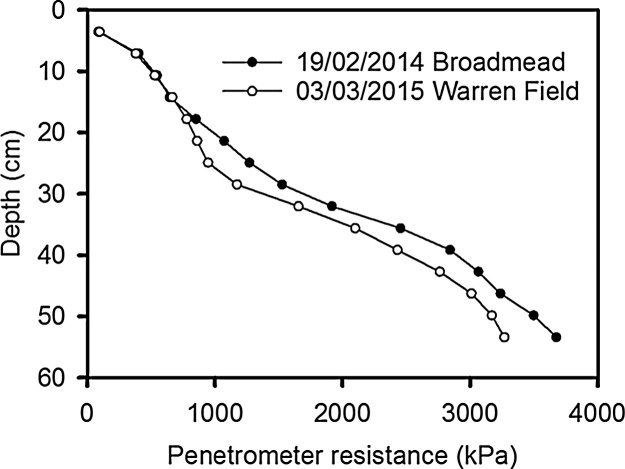
Soil strength with depth in the two field sites over the two years in early spring. These measurements were made before any soil drying. These data are the means of the four replicate plots and five penetrometer measurements were made in each plot.

The soil penetrometer resistance profiles before any water uptake were similar for each year (Fig. 1) and both show that even in the absence of any soil drying (i.e. the soil profile was at field capacity) penetrometer resistance increases with depth.

### 3.2 Soil water content

**Fig. 2:**
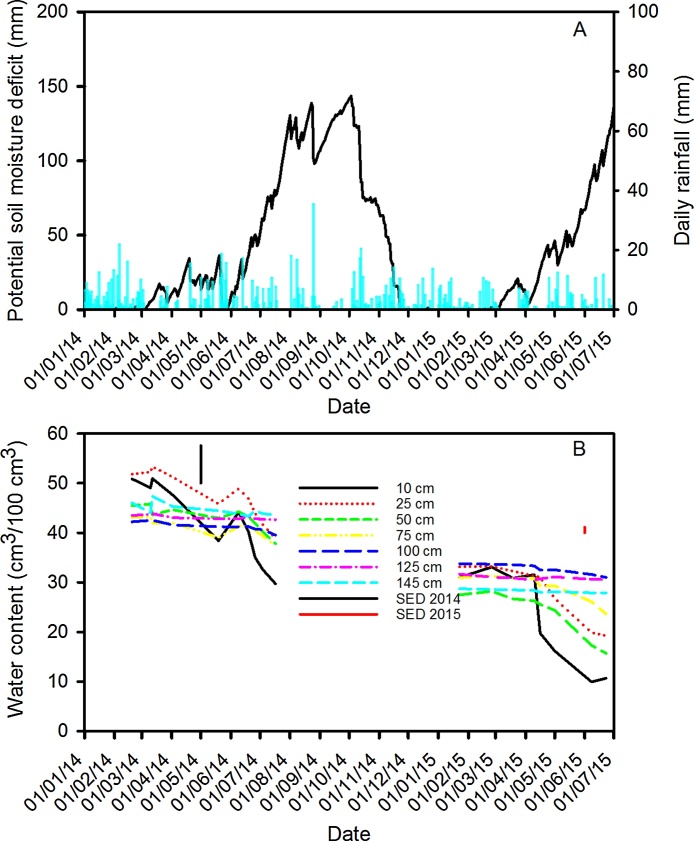
Daily rainfall and potential soil moisture deficit (A) and soil water content at different depths in 2014 and 2015 (B) . These are the means taken across the different wheat lines (data from the fallow plots were excluded). In 2015 there was no effect of wheat line on soil water content profile, but a significant (P < 0.05) effect was observed in 2014 (see Fig. 3). Potential soil moisture deficits (A) for the duration of the experiment calculated from meteorological data with the Penman Monteith method. Redrawn from Whalley et al. (2017), where it is presented as supplemental data.

**Fig. 3:**
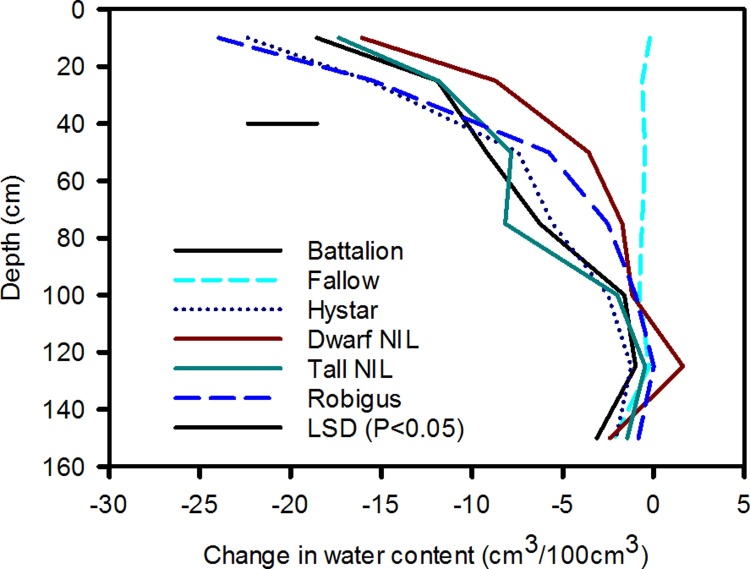
Change in water content with depth between 19/02/2014 and 17/07/2014 for the wheat lines studied in 2014 as a function of depth. Data for the fallow plot is also shown. In 2015 there was no effect of wheat line on soil water content profile and the main effect of depth on soil drying for both 2014 and 2015 is shown in Fig. 4.

**Fig. 4:**
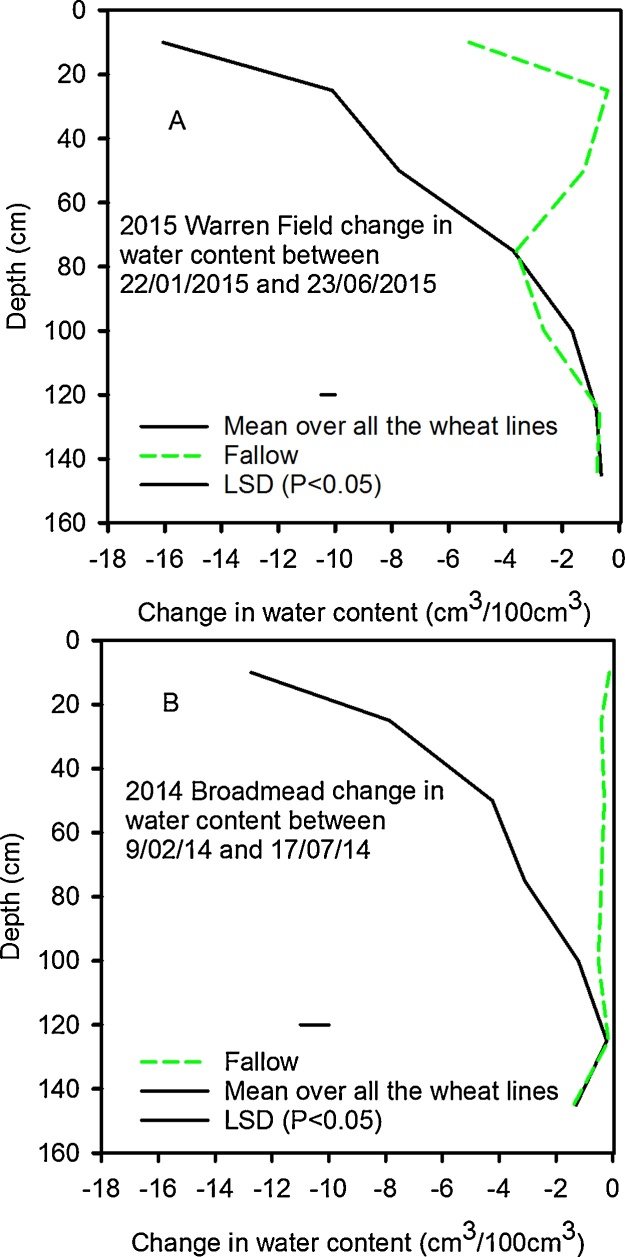
The change in soil water content between 19/02 and 17/07 in 2014 and 22/01 and 23/06 in 2015 as a function of depth. For the sown plots these data are the means taken across the different lines. Data for the fallow plots are also shown. The main effect of wheat on soil drying profile is shown in Fig. 3 for 2014, but in 2015 only the main effects and interactions between time and depth (Fig. 2) were significant. In both years the effect of depth was significant (P < 0.001).

**Fig. 5:**
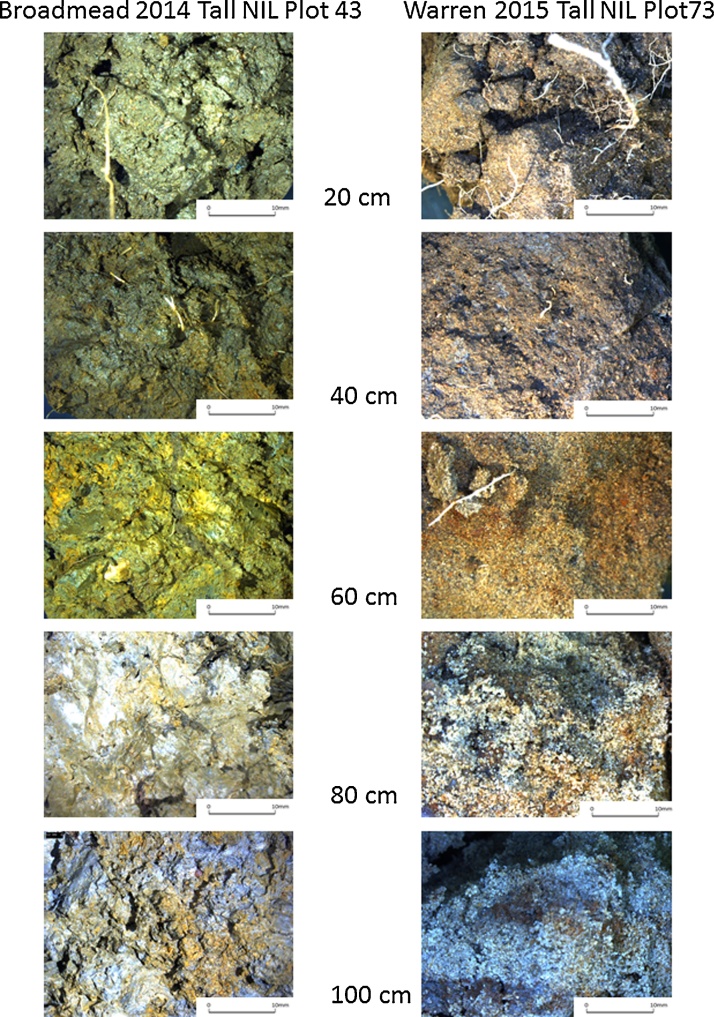
Examples of photographs taken during the core-break method from Broadmead (2014) and Warren Field (2015) for the Tall NIL. Comparison of the soil showed that Broadmead had uniformly deep clay whereas Warren Field had a sand dominated subsoil. The scale bar is 10 mm in length.

Soil moisture deficit (Penman-Monteith) and rainfall were consistent with the pattern of soil water content measured with the neutron probe (Fig. 2). These data show that there were different temporal patterns of soil drying in 2014 and 2015. In 2015 the soil profile remained wet until mid-April when there was a period of intense soil drying, whereas in 2014 soil drying began earlier in the season. The lower water contents in 2015 compared with 2014 are probably due to the higher sand content in deeper layers in Warren Field (2015) compared with Broadmead (2014). In 2014 wheat line had a significant effect on the soil drying profiles (Fig. 3). Here data are plotted as the difference in water content relative to a date in winter when the soil was assumed to be at field-capacity. These data are compared with data from the fallow plots and differences relative to the fallow plots are assumed to be due to water uptake by roots. The Dwarf NIL was the least effective at drying the soil. At depths of approximately 50–80 cm, the Tall NIL, Hystar Hybrid and Battalion dried the soil the most. In 2015, neutron probe measurements did not show any significant effects of wheat line on soil drying. In 2014 the soil was dried to greater depth than in 2015 (Fig. 4). In 2015, a reduction in water content on the fallow plots (Fig. 4), between depths of 60 and 120 cm, is most likely due to drainage of soil water, which is consistent with a sand dominated subsoil on Warren Field. In the surface layer of Warren Field between 20 and 50 cm, the water does not drain. This is likely due to the phenomenon of a perched water-table which can occur when fine soil is on top of coarse soil (Campbell, 1985), as is the case for Warren Field (see later; Fig. 5). The limited drainage of fallow plots on Broadmead field is also consistent with a profile composed of a silt-clay loam in the 0–100 cm layer. In the absence of matric potential data, the use of the fallow plots is helpful to discriminate between drainage and soil drying by roots. The estimated potential soil water deficits (Fig. 2) showed that 2015 was drier than 2014, thus wetter soil in 2015 is likely to be related to lower water uptake by wheat or impaired drainage in the 0–40 cm layer. Perched water tables are common in combinations of fine textured top soil and coarse textured subsoil, as found in Warren Field.

### 3.3 Soil temperature

Table 2 shows the accumulated thermal time, using a base temperature of 0 °C, between the sowing date and root sampling in each of experiments, estimated using the data from the meteorological station, because the thermistors were not installed during the initial growth period. Although the temperature data is from a meteorological station, the data provided an accurate estimate of temperature beneath the wheat plots (data not shown).

**Table 2:** Accumulated thermal time between the sowing date and root sampling in degree days.

	Accumulated thermal time (degree days)		
Growth Season	Depth (cm)		
	10	50	100
2013/2014	2233	2276	2271
2014/2015	2679	2783	2424

### 3.4 Root depth profiles

**Fig. 6:**
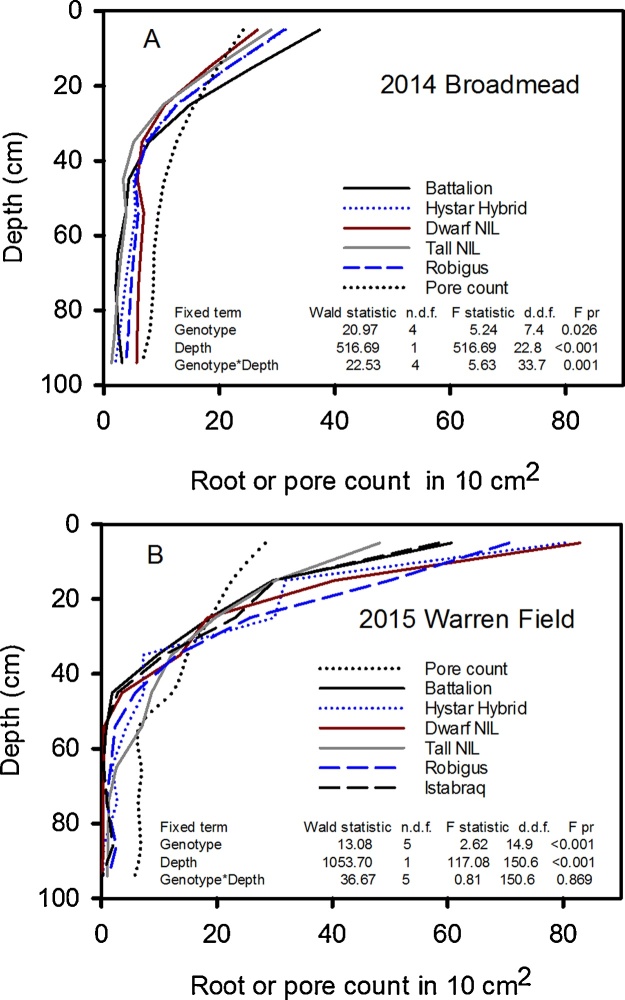
The distribution of roots with depth in 2014 and 2015. These data were obtained with the core break method. A summary of the statistical analyses is also shown. The cores were 10 cm long and the number of roots counted on 10 areas in each 10 cm^2^ is numerically equivalent to root length density in cm/cm^3^. Output from REML analysis is shown and this applies only to the root data. There data were square root transformed before analysis, so LSDs cannot be presented. The only significant effect of pore count was that of depth in both 2014 and 2015.

Example soil images with depth at 20 cm increments indicate the clay subsoil at Broadmead, and the sand layers at depth at Warren Field (Fig. 5). Root numbers on ten 1 cm^2^ areas were counted on the upper face of the cracked core and these data are plotted as a function of depth along with the counts of pores greater than 0.7 mm in diameter (Fig. 6). As the cores were 10 cm in length, the total root count in 10 cm^2^ is numerically equivalent to root length density in cm/cm^3^, assuming that the roots are vertical. The vertical root distribution differed between wheat lines, as indicated by a significant line x depth interaction for transformed root numbers in 2014 (P = 0.001) but not in 2015 (P = 0.87). Thus, the decrease in root number with depth is represented by separate slopes for each cultivar in 2014, while in 2015, the same slope across all cultivars was sufficient (see Table 3). Moreover, wheat line had a significant main effect in 2014 (P = 0.026) and 2015 (P < 0.001) on the number of roots counted. In 2014, Battalion had more roots in the surface layer than the other wheat lines (37.4 compared with 31.5 for Hystar Hybrid), while the Dwarf NIL had the greater number of roots at depth (5.7 compared with 3.1 for Battalion).

**Table 3:** Slopes for 2014 for each line and the common slope for 2015 on the square root scale (the rate of change per cm of depth). These data show that the root length distribution with depth in 2014 depended on wheat line, but not in 2015. In 2014 the smallest negative slope for the Dwarf NIL, shows that it had the deeper rooting habit (see also Fig. 6). The more negative slope for 2015 in comparison the slopes for 2014 reflects the shallower rooting in 2015. In 2014 a single standard error applies to all of the wheat lines.

Wheat line	Slope	Standard error
Battalion	−0.05314	
Hystar Hybrid	−0.04477	
Dwarf NIL	−0.02588	0.004450
Tall NIL	−0.04584	
Robigus	−0.03709	

2015	−0.07888	0.003728

Wheat line did not significantly (P = 0.072 in 2015 and P = 0.212 in 2014) affect the number of soil pores counted, nor was there any interaction between wheat line and depth affecting pore count (P = 0.898 in 2015 and P = 0.098 in 2014). While there was no reason to expect that wheat line should affect pore count, these data gave us confidence that the method used to break the cores did not result in empty root channels due to roots being pulled out of the soil, to the extent that the inferences drawn on pore count data are affected. We also noted that the distribution of pore counts with depth were similar on both Broadmead in 2014 and Warren Field in 2015.

**Fig. 7:**
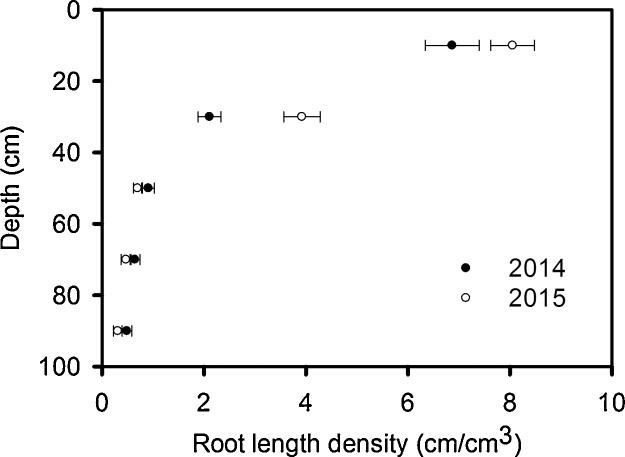
The root length density data for 2014 and 2015 determined from the root washing method. There was no significant effect of wheat line in either 2014 or 2015. The SE of the means is shown for 3 replicates in 2014 and four replicates in 2015.

Wheat line did not significantly affect root length density determined from washing roots out of the 10 cm long cores in either 2014 or in 2015 (Fig. 7). However, data from both root washing and core break methods (compare Fig. 6, Fig. 7) showed similar differences between years (i.e. shallower rooting in 2015 and deeper rooting in 2014).

### 3.5 Shoot growth

**Fig. 8:**
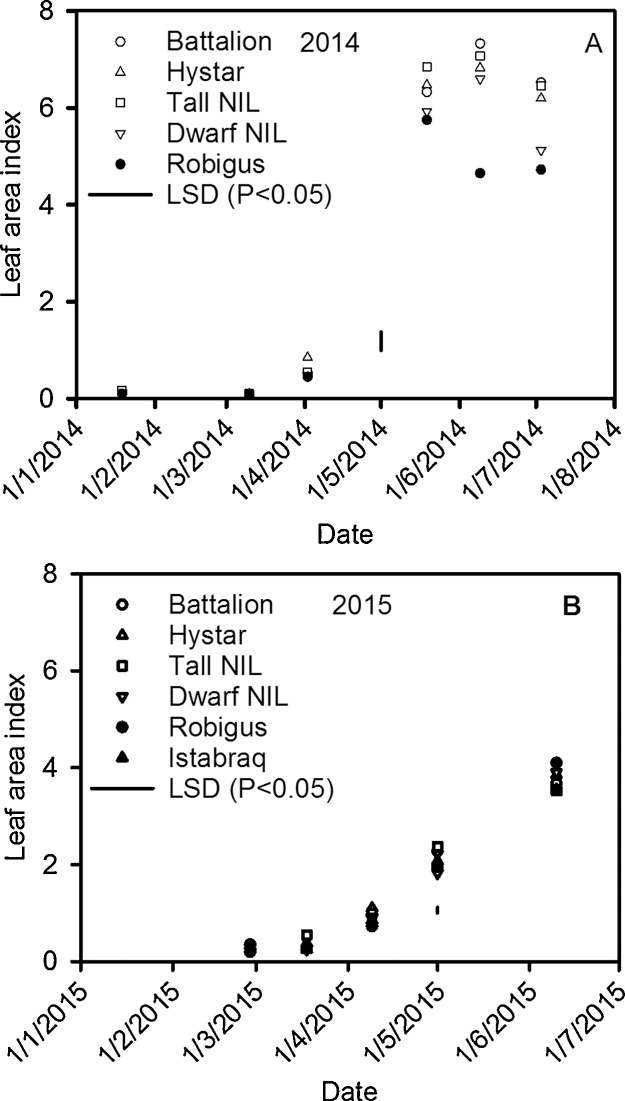
Leaf area index estimated with a ceptopmeter. The data plotted are the means from four replicates.

Leaf area index (LAI) of all the wheat lines were similar within each year but differed between 2014 and 2015 (Fig. 8). In 2015 LAI increased over time, but at a slower rate than in 2014. In 2014, LAI peaked in mid-June for all genotypes except Robigus, for which LAI had already started to decline towards the end for the season related to senescence. Robigus is susceptible to yellow rust (*Puccinia striiformis*) and disease pressure was high in 2014. In 2015 we measured crop height (Table 4). Although we did not measure crop height in 2014, these heights reflect our visual observation, especially the small height of the Dwarf NIL in comparison with all other lines. Except for the Dwarf NIL, the yield was higher in 2014 than 2015 (Table 5). The yields of the commercial wheats are typical of those grown in the UK.

**Table 4:** Crop height measured on 19 June 2015.These data represent the mean of four replications.

Wheat line	Mean crop height (cm)	Standard error
Battalion	73.1	0.32
Hystar Hybrid	80.3	1.32
Istabraq	80.3	0.51
Dwarf NIL	44.4	0.61
Tall NIL	80.8	1.44
Robigus	74.4	1.43

**Table 5:** Yield data for 2014 and 2015. These data represent the mean of four replications. The SED values for 2014 and 2015 were 0.54 and 0.89 t/ha (66 df) respectively with P < 0.001 in all cases.

Wheat line	Year	
	2014	2015
Battalion	12.17	9.10
Hystar hybrid	12.28	10.86
Istabraq	12.65	9.67
Dwarf NIL	4.15	5.46
Tall NIL	10.87	9.27
Robigus	7.96	6.52

## 4 Discussion

### 4.1 Effect of soil conditions on root elongation

Wheat plants grown in rhizotrons can have very high root length densities at depth, due to differences in root morphology between laboratory and field-grown plants (Gao et al., 2016, Manschadi et al., 2008). In contrast, when the soil cores were taken from the field to estimate root distribution and the wheat was at heading stage (when root dry weight is reportedly at or near its maximum and root length distribution with depth has reached a steady state − Gregory et al., 1978a), root length density decreased rapidly with depth (Fig. 6, Fig. 7). Indeed, empirical root length density models are based on this relationship (e.g. Gerwitz and Page, 1974, Fan et al., 2016) and similar root length distributions have been reported in field grown winter wheat (Gregory et al., 1978a, Lupton et al., 1974, White et al., 2015). At depths below 40 cm when the soil is well-watered (Fig. 1), or below 20 cm following soil drying by transpiration, penetrometer resistance exceeds 2500 kPa (data not shown), a value associated with very low rates of root elongation (Bengough and Mullins, 1991, Taylor et al., 1966). In soil layers deeper than 35 cm, the root length density is greatly reduced compared with the surface layer (Fig. 7). The reduction in root length density with depth is likely a response to increasing penetrometer resistance (Fig. 1). At a depth of approximately 35 cm, the numbers of root and pores are comparable (Fig. 6). In deeper layers there are more pores than roots whereas in the surface layers there are more roots than pores.

The relationship between rooting and soil pores at depth is hard to interpret. One definitive result is that at depth there are a large number of empty pores. An extreme example is for one of the Hystar Hybrid cores where at a depth of 95 cm there was 1 root and 20 empty pores. While it is difficult to be certain, our data appear to support the conclusion of White and Kirkegaard (2010) that at depth roots are confined to pores. Since roots can expand in pre-existing pores to fill them, inspection of photographs is inconclusive and depends on the threshold size used to define pores. We chose a pore size of 0.7 mm because most wheat roots are smaller than this (e.g. Jin et al., 2015). However, roots can elongate in pores smaller than the nominal root diameter with relative ease, because their elongation rate is most sensitive to axial pressure and not the radial confinement that would be applied by pores (Bengough, 2012, Bengough and Mackenzie, 1994, Jin et al., 2013). Thus, observing a root that is in intimate contact with soil cannot be taken as evidence of root penetration by deformation. In our view, field studies alone are unlikely to determine whether deep rooting can be achieved by soil deformation, however, it does seem improbable.

A reduction in the pore density with depth was also found in Australia (White and Kirkegaard, 2010) for pores greater than 0.2 mm in diameter and Germany (Athmann et al., 2013) for pores greater than 5 mm in diameter. In addition to fewer pores at depth, an incomplete level of pore occupation by roots at depth (Fig. 6) contributes to a sparse root length density in deep soil layers. In this respect our data are consistent with those published by White and Kirkegaard (2010) for Australian grown wheat (5% of pores contained roots at a depth of 1 m) and for barley grown in Germany (Athmann et al., 2013) (85% of pores contained roots at a depth of 1 m).

To compare the effect of temperature on root growth, accumulated thermal time is useful (Barraclough and Leigh, 1984; Table 2). Greater thermal time was accumulated at soil depths of 10, 50 and 100 cm for roots harvested in 2015 compared with 2014. While these temperature effects may explain the greater amount of surface rooting in 2015 compared with 2014 (Fig. 7), lower soil temperatures at depth cannot account for the lower counts of deep roots in 2015.

In each year, soil strength profiles determined with a penetrometer were similar before any soil drying. Furthermore, soil temperature profiles do not appear to explain fewer deep roots in 2015 compared with 2014. Hence it seems likely that the large differences in the root length distribution between 2014 and 2015 (Fig. 6) are related to differences in the saturation of the soil profile (Fig. 2). In 2015 there was limited soil drying until the beginning of April, and the shallower rooting in 2015 compared with 2014 may be related to limited oxygen availability due to higher levels of soil saturation (Gliński and Stępniewski, 1986, Blackwell and Wells, 1983). Between sowing and 1 April in 2014 and 2015, the accumulated rainfall on the sites was 490 and 374 mm respectively, apparently excluding weather as an explanation for the wetter conditions in 2015. The most likely explanation for the wetter soil profile in early 2015 is differences in drainage between the adjacent fields.

When grown in the field (Fan et al., 2016, Gao et al., 2016, Thorup-Kristensen et al., 2009) or in rhizotrons (Jin et al., 2015, Manschadi et al., 2008), wheat roots can grow to depths of 1 m or more, as observed in 2014. However, shallow rooting depths (< 60 cm) for wheat, similar to our 2015 data, are also commonly reported when a water table is present (e.g. Xue et al., 2003, Brisson et al., 2002). Wheat root growth is greatly affected by the presence of a water table, and a shallow water table (approximately 60 cm deep) limited root growth below 40 cm at 38 days after sowing (Zuo et al., 2006). Measurements of water potential would have been needed to confirm the presence of a perched water table in our 2015 experiment, however, the greater sand content of the sub-soil makes this likely. Furthermore, soil water content data from the fallow plots in Warren Field (Fig. 4) clearly shows limited drying of the of clay rich layer between 20 and 50 cm and drainage of deeper layers between 60 and 120 cm, dominated by sand. Impeded drainage of fine textured soils overlying drained coarser soil, to give a perched shallow water table, is consistent with the physics of drainage (Campbell, 1985).

### 4.2 Genotypic effects

We found a significant interaction between wheat line and depth in 2014, but not in 2015 (Fig. 6, Table 3) with respect to rooting density. In 2014 the genotypic differences in root length distribution are correlated with soil water measurements in the surface layer. Battalion is one of the most effective wheats at drying the soil (Fig. 3) and has more surface roots than the other lines (Fig. 6). In contrast, the Dwarf NIL had the fewest surface roots and was less effective at drying the surface soil. In the top 25 cm, the root number explained 76% of the variation in soil drying measured with the neutron probe (P < 0.003). In 2015, genotype had no significant effect on either water uptake or on root distribution. Root count data shows that there were more roots at depth in 2014 than 2015 (Fig. 6), which is supported by root length data (Fig. 7) and the finding of deeper soil drying in 2014 (Fig. 4). These data support the use of soil water content measurements as proxy for root length as previously discussed (Wasson et al., 2012). However, at depth there was limited water uptake from any of the studied wheat lines (see also Whalley et al., 2017).

In the surface layers the number of roots was greater than the number of pores (Fig. 6). This suggests root proliferation in the non-structured pore space, in this case soil where pores are smaller than 0.7 mm in diameter. In 2014, when there was a significant genotype-depth interaction (P = 0.001), Battalion had the greatest number of shallow roots (0–20 cm). In laboratory studies (Whalley et al., 2013), Battalion had more roots than all the other wheats studied here (although Hystar Hybrid was not studied). Battalion was also better at penetrating strong layers, which has been confirmed by subsequent investigations (unpublished data of Whalley), which might explain why it has a greater number of surface roots in comparison with the other lines. It is important to note that in the surface layer, the penetrometer resistance (Fig. 1) in well-watered soil (i.e. between sowing and early-March 2014 and mid-April 2015 in the respective years; Fig. 2) is low. This is consistent with the observation that all the wheat lines studied, in both years, have higher root numbers in these surface layers of mechanically weak soil.

While the numbers of roots deeper than 60 cm for all wheats is small in 2014, the Dwarf and Tall NILs were the extremes, with the Tall NIL having the fewest deep roots. One possible explanation for the deeper rooting of the Dwarf NIL compared to the Tall NIL in 2014 (Fig. 6) is that the location of pores by roots is related to the number of roots. At 95 cm, 85% of pores were filled by Dwarf NIL roots but only 20% for Tall NIL in 2014 (2014, Fig. 6) using a 0.7 mm pore size as a threshold. Laboratory studies have shown that Rht-B1a (*Tall NIL*) has fewer root axes compared with Rht-B1c (*Dwarf NIL*) when grown in a low impedance environment (Coelho Filho et al., 2013). However, when the substrate impeded growth, the number of roots was similar for both NILs, and indeed in the surface layer of the field both NILs have a similar number of roots (Fig. 6a). A greater number of roots might explain a greater likelihood of pore location (Hewitt and Dexter, 1979) otherwise some biological mechanism allowing roots to locate pores would be required. Our data appear to agree with those of Miralles et al. (1997), who also found that dwarf wheat had greater rooting mass and length in comparison with taller wheats.

The greater root density at depth of the dwarf wheat was not reflected in greater water uptake, possibly because even for this wheat the root density at depth was too low to allow all the available water to be accessed (Gregory et al., 1978a, Gregory et al., 1978b). Although the leaf area index is not affected by dwarfing (Fig. 8), the shorter canopy of the dwarf wheat (compared with all the other wheats we studied) may have confounded comparisons of water uptake (Table 4). In 2014 and 2015 at depths greater than 40 cm, the root length density of all lines does not change greatly with depth (Fig. 6, Fig. 7). However, in the same depth interval (40 cm to 95 cm) water uptake by roots depends strongly on depth (compare Fig. 3, Fig. 4 with Fig. 6, Fig. 7). It seems that in this region, the ability of the roots to dry soil is only weakly related to root length density. While capillary rise could partly explain limited soil drying between 40 cm and 95 cm, it is unlikely to be the complete explanation, given the limited drying of the fallow plot due bare-soil evaporation (Fig. 3). It is likely that with increasing depth, a greater proportion of roots are found in pores and hence root orientation varies with depth. This is important in determining water uptake because the ability of roots to dry soil depends on their geometrical arrangement in soil, irrespective of soil to root contact (Passioura, 1991). Passioura (1991) demonstrated that vertical roots, in vertical cylindrical pores, which probably occur increasingly in the deeper layers, provide the least effective geometry at enabling roots to dry soil. A further complication is poor contact between roots in pores and the bulk soil (e.g. White and Kirkegaard., 2010). It is widely reported that flux of water through bulk soil does not appear to explain poor water uptake (Deery et al., 2013a, Deery et al., 2013b) and radial hydraulic resistance between the root and soil is thought to limit water uptake (e.g. Herkelrath et al., 1977).

In contrast to root length densities estimated from the core break method, root washing showed no genotypic effects in either 2014 or 2015 (Fig. 7). The main effect of year was similar with data obtained from both methods (Fig. 6, Fig. 7), although magnified in the data from the core-break method. It is difficult to be certain why the core break method discriminated differences between wheat lines with respect to root growth in 2014 while the root washing method did not. It is possible that some roots were lost during root washing, rendering this method less sensitive. Alternatively, the root core break method may have been less effective at detecting horizontal roots branched from the vertical axis.

### 4.3 Relationship between yield, shoot growth and rooting

The lower yield in 2015 (Table 5), except for the Dwarf NIL, is consistent with a smaller leaf area index (Fig. 8) and a shallower root system (Fig. 6, Fig. 7) in 2015. Although the dwarf phenotype of Rht-B1c (*Dwarf NIL*) was observed (Table 4), there was minimal effect on leaf area index, although in 2014 it was somewhat smaller than the Tall NIL and all other wheats (except Robigus which had yellow rust). Although the Tall NIL does not contain the dwarfing gene, in a Mercia background the Tall Rht allele is comparable in height to the commercial semi-dwarf lines (Table 4). Yield is more closely related to plant height than to the particular allelic dwarfing nature (Addisu et al., 2010). The optimum plant height for a maximum yield is in the range 70–80 cm (Addisu et al., 2010) which is comparable to the height of the wheats studied, with the exception of the extreme dwarf NIL. The effect of Rht genes on yield is related to relatively complex pleiotropic effects on spike fertility, grain number and grain size; including an interaction between grain number and size (Youssefian et al., 1992, Flintham et al., 1997). This is consistent with our observation the extreme dwarf NIL has sufficient rooting (Fig. 6) and leaf area (Fig. 8) to capture water, nutrients and light when compared with the other wheat lines in this study.

## 5 Conclusions

We present evidence to support the hypothesis that the general and well-documented shape of the relationship between root length density and soil depth in UK grown winter wheat is related to the increase in soil strength with depth as well as the distribution of root-sized biopores with depth and/or the ability of roots to locate them. In the two years of this study, effects of the soil environment on root length distribution were greater than genetic effects and this was most likely related to soil saturation. In the drier of the two years, there was genotypic variation in rooting depth. However, the greater root length at depth in the dwarf NILs suggests that deep rooting is not simply related to plant height.

## Appendix A

Genstat code to fit spline function to root count data

180 "

−181 fit parsimonious model

−182 "

183 vcomp [fixed = Genotype*xd; spline = xd] Block/plots/layer; con = pos

184 vstruct [term = Block.plots.layer] model = ar; order = 1; factor = layer; het = outside

185 reml [prin = model,moni,comp,devi,wald; maxcyc = 50] sTotal_R

sTotal_R is the square root transformed numbers of roots at each layer for each Genotype. xd is a variable representing the depth in cm at which the measurements were made. The 3 commands specify 1) on 183 the statistical model for the fixed and random components of the residual maximum likelihood model, 2) on 184 the kind of spatial correlation between the levels of the factor, layer, and allows for additional variability at each layer (het = outside) and 3) on 185 what summary statistics and other output are presented in the analysis. Note that the modelling process is iterative and so this represents the final model and includes the interaction between Genotype and depth when it is statistically significant.
